# Agarwood Alcohol Extract Ameliorates Isoproterenol-Induced Myocardial Ischemia by Inhibiting Oxidation and Apoptosis

**DOI:** 10.1155/2020/3640815

**Published:** 2020-07-09

**Authors:** Canhong Wang, Deqian Peng, Yangyang Liu, Zhangxin Yu, Peng Guo, Jianhe Wei

**Affiliations:** ^1^Hainan Provincial Key Laboratory of Resources Conservation and Development of Southern Medicine, Key Laboratory of State Administration of Traditional Chinese Medicine for Agarwood Sustainable Utilization, Hainan Branch Institute of Medicinal Plant Development, Chinese Academy of Medical Sciences and Peking Union Medical College, Haikou 570311, China; ^2^School of Pharmacy, Hainan Medical College, Haikou, Hainan 571199, China; ^3^National Engineering Laboratory for Breeding of Endangered Medicinal Materials, Key Laboratory of Bioactive Substances and Resources Utilization of Chinese Herbal Medicine, Institute of Medicinal Plant Development, Chinese Academy of Medical Sciences & Peking Union Medical College, Beijing 100193, China

## Abstract

Agarwood is a traditional medicine used for treating some diseases, including painful and ischemic diseases. This study was carried out to investigate the potential cardioprotective effect of the whole-tree agarwood-inducing technique-produced agarwood alcohol extract (WTAAE) on isoproterenol- (ISO-) induced myocardial ischemia (MI) in rats and explore the underlying molecular mechanisms. Compared to the MI group, WTAAE pretreatment significantly improved ST wave abnormal-elevation, mitigated myocardial histological damage; decreased creatinine kinase (CK), lactate dehydrogenase (LDH), alanine transaminase (ALT), and aspartate transaminase (AST) levels; reduced hydrogen peroxide (H_2_O_2_) and lipid peroxide (LPO) production; and increased total antioxidant capacity (T-AOC) and catalase (CAT) activities. Moreover, agarwood alcohol extracts (AAEs) markedly enhanced the mRNA levels of Nrf2-ARE pathway, and Bcl-2 reduced the apoptotic Bax family mRNA expressions. In addition, the effect of WTAAE was greater than that of wild agarwood alcohol extract (WAAE) and burning-chisel-drilling agarwood alcohol extract (FBAAE). All of these data indicate that WTAAE exerted the protective effects of MI, and its mechanism was associated with upregulating Nrf2-ARE and suppressing Bcl-2 pathways.

## 1. Introduction

Agarwood, the genus *Aquilaria* and the family Thymelaeaceae, is a valuable nontimber forest product [1]. It is well known that agarwood is a precious traditional Chinese medicine for centuries to treat several diseases, including heart diseases such as angina pectoris [2–4]. Subsequently, the different parts of agarwood extracts were found to possess alleviating pain and antioxidant and other biological activities, which support its folkloric and clinical use for some diseases related to painful conditions, such as angina, trauma, gastric ulcer, enteritis, and coughs [5]. At present, the extracts, essential oil, and main compounds from agarwood have exhibited a wide array of pharmacological properties such as laxative [6], antinociceptive [7], antineuroinflammatory [8], neuroprotective [9], antioxidant [10], anti-inflammatory [11], and antitumor activities [12]. According to its traditional efficacy and modern application, we boldly hypothesize that agarwood could be used to treat the myocardial ischemia (MI) injury. However, there is still no available study evaluating the effects of WTAAE on MI rats and its mechanism by modern pharmacological studies.

Cardiovascular disease is one of the major health problems worldwide. Extensive research studies focused on studying cardioprotective therapies throughout the last decade, among which MI attracts more attention attributes to its complex pathophysiological process [13–15]. The main culprit in MI is that the coronary blood supply does not meet the demand of cardiomyocytes, which leads to the rapid development of myocardial necrosis [16]. Usually, ischemia occurs when there is no blood flow through the myocardial tissue, which leads to the imbalance of supply and demand with oxygen, and finally results in the dysfunction or injury of tissue, named MI [17]. The pathogenesis of MI involves various mechanisms including inflammatory responses [18], accumulated free radical [19], and cell apoptosis [20]. The treatment of MI has been a vital problem, because there is still no advanced and effective therapeutic regimen [21]. Traditional Chinese medicine (TCM) is a unique medical field that encompasses pathogenesis, diagnosing diseases, and drug therapy [22]. Over the past decades, TCM has shown a potential therapeutic effect on cardiac diseases and shown positive results in clinical trials [23]. Furthermore, a previous study revealed that the WTAAE had provided significant myocardial protection [24]. Therefore, more clearly, experimental studies need to explore the potential role of TCM including agarwood in the treatment of MI.

The present study is designed for the first time to investigate the potential cardioprotective effects of WTAAE on ISO-induced MI in rats and explore its possible mechanisms, including antioxidative and antiapoptotic effects. Besides, we aimed to compare the effects of different agarwood alcohol extracts (AAEs) against MI. This paper provides the experimental evidence for further research and clinical treatments of the protective mechanisms of agarwood against myocardial diseases.

## 2. Materials and Methods

### 2.1. Reagents

Isoproterenol (ISO) was obtained from Harvest Pharmaceutical Co., Ltd., (Shanghai, China). The myocardial enzymes, such as creatinine kinase (CK), lactate dehydrogenase (LDH), alanine transaminase (ALT), and aspartate transaminase (AST), were purchased from Biosino Bio-Technology and Science Inc., (Beijing, China). All biochemical indicator kits, cell death kit (CDK), hydrogen peroxide (H_2_O_2_), lipid peroxide (LPO), total peroxide capability (T-AOC), and catalase (CAT) were purchased from Jiancheng Biotech Co., (Nanjing, China). The primers of Nrf2-ARE and Bax/Bcl-2 signaling pathways were synthesized by Sangon Biotech (Beijing, China). Anhydrous alcohol and other chemicals procured were of analytical grade.

### 2.2. Materials

The whole-tree agarwood-inducing technique [1] (patent number: ZL201010104119.5) producing agarwood, wild agarwood, and burning-chisel-drilling agarwood was purchased from Guangdong, China, and authenticated by Researcher Jian-He Wei, Institute of Medicinal Plant Development, Chinese Academy of Medical Sciences and Peking Union Medical College, Beijing, China. Dried and powered agarwood (1000 g) produced by whole-tree agarwood-inducing technique was soaked in 95% alcohol (5 L) for 2 h and refluxed for 1 h three times and combined with the solution. The resulting solution was evaporated in vacuo to get the dark brown EtOH extract (140 g, 14%) and the conserved at −20°C. The EtOH extracts of wild agarwood and burning-chisel-drilling agarwood were produced by the same method with leaching rate 10.5% and 14%, respectively.

### 2.3. Animals

Adult male Sprague–Dawley rats (180–220 g) were purchased from Vital River Laboratory Animal Technology Co., Ltd. (Beijing, China) and were kept on a 12 h light/dark cycle with controlled temperature (20–24°C) and humidity (50–70%). Mice, with free access to food and water, were housed for 5 d prior to the experiments. Animal care and experimental protocols used in this study were approved by the Institutional Animal Care and Use Committee at the Institute of Medicinal Plant Development, Chinese Academy of Medical Sciences.

### 2.4. Animal Experiments

Rats were randomly divided into seven groups with eight mice in each group: normal group, ISO group, WAAE (equivalent of crude drug 2.84 g/kg) group, FBAAE group (2.84 g/kg), and WTAAE groups (0.71, 1.42, and 2.84 g/kg). Rats in the normal and model groups were orally administered with distilled water (20 mL/kg, 1 time/day). Rats in treatment groups were orally administrated with the AAEs (20 mL/kg, 1 time/day), respectively. After 7 consecutive days of pretreatment, rats were intoxicated with ISO except for the control group (2 mg/kg and 1 mg/kg, respectively) by subcutaneous injection on 2 consecutive days.

### 2.5. Electrocardiogram (ECG)

ECG test was conducted in anesthetized rats after finishing ISO injection. The needle electrodes were linked to the right arm, left arm, and left leg in rats. ECG and ST elevation were recorded by the BL-420S Biological Function Experiment System.

### 2.6. Histopathological Examination of Myocardium

Hearts were removed and fixed in 4% paraformaldehyde, dehydrated, and paraffin embedded. Then the samples were sectioned at 5 *μ*m (Leica DMR, Germany). For histological analysis, sections were stained with hematoxylin-eosin (HE) by standard techniques and examined by light microscopy (Nikon, Tokyo, Japan) at 200x magnification.

### 2.7. Determination of Myocardial Apoptosis

Myocardial apoptosis was determined by terminal deoxynucleotidyl transferase-mediated dUTP-biotin nick end labeling (TUNEL) staining. TUNEL staining was performed by using In Situ Cell Death Detection Kit. Heart tissue samples for the determination of myocardial apoptosis were dewaxed in xylene for 10 min, twice, and 100%, 95%, 90%, 80%, and 70% ethanol hydrated for 2 min, respectively. Samples were washed 3 times using PBS, 5 min each time, and 20 g/mL of dNase-free protease K was added and kept for 30 min at 20–37°C. Samples were washed 3 times using PBS, 5 min each time, and sealed immersing in 3% H_2_O_2_ methanol solution at room temperature for 10 min. Samples were washed twice using PBS, 5 min each time. 50 *μ*L TdT enzyme reaction solution was added to the sample and incubated in darkness at 37°C for 60 min. Samples were washed 3 times using PBS and observed under a fluorescence microscope. Further stained with DAB, 50 *μ*L antifluorescein antibody was first added and incubated in darkness at 37°C for 30 min. Samples again were washed 3 times using PBS, 5 min each time. 50 *μ*L DAB working fluid was added and incubated at room temperature for 30 min. Dyed samples were observed and photographed under an optical microscope.

### 2.8. Levels of Myocardial Enzymes in Serum

Myocardial cellular damage was evaluated by measuring myocardial enzymes in serum. The levels of CK, LDH, ALT, and AST were measured according to the manufacturer's instructions.

### 2.9. Determination of Oxidation Index Level in Plasma

Blood was collected and anticoagulated with EDTA-K2. The plasma was centrifuged at 3000 rpm at 4°C for 15 min. The plasma was collected and stored at −80°C. LPO, H_2_O_2_, T-AOC, and CAT were detected using special kits following the manufacturer's instructions.

### 2.10. Analysis mRNA Levels of the Genes Involved in Antioxidation and Apoptosis

The mRNA levels of the genes involved in antioxidation and apoptosis are determined by a real-time quantitative PCR. Total RNA in heart tissues from myocardial ischemia injury rats and AAEs (2.84 g/kg) groups were extracted and reverse-transcribed into cDNA for 30 min at 42°C and 5 min at 85°C using the RT kit. The cDNA was amplified using a RT-PCR system by PCR Supermix kit, using the Power SYBR Green PCR Mix. Samples were cycled 40 times using the CFX96 Touch TM PCR System (BioRad, Hercules, CA, United States). Conditions were as follows: 5 min at 95°C followed by 40 cycles of 15 s at 95°C, 34 s at 60°C, and 40 s at 72°C. Cycle threshold (CT) was calculated under default setting for real-time sequence detection software [22]. At least three independent biological replicates were performed to check the reproducibility of the data. Sequences of the Nrf2, HO-1, GST, Bax, Bad, Bik, Bcl-2, and *β*-actin and the gene-specific primers are listed in Table 1.

### 2.11. Statistical Analysis

Data were expressed as mean ± SD for ten animals in each group and statistically evaluated with SPSS17 software (Chicago, IL, USA). Differences between the groups were performed by one-way (ANOVA) and Tukey's post hoc test (TTEST). *P* values less than 0.05 were considered to indicate statistical significance.

## 3. Results

### 3.1. Effect of AAEs on the ECG

**Figure 1:**
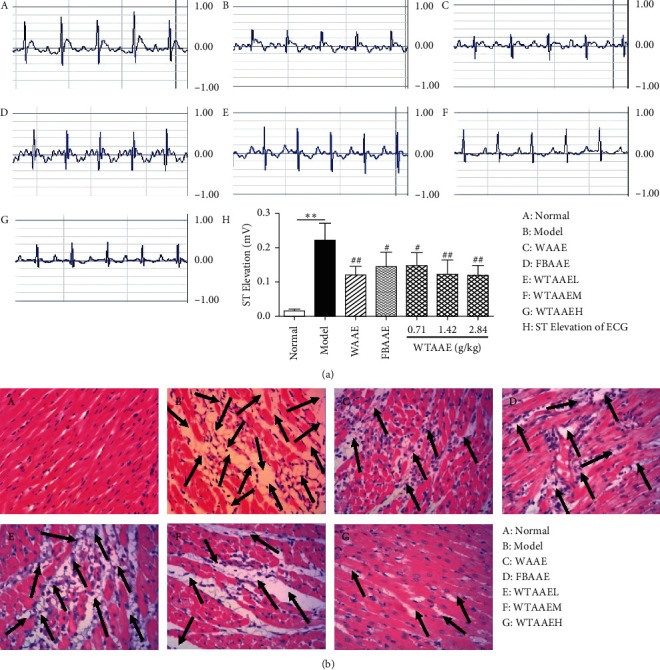
Effects of orally administered AAEs on the electrocardiogram (ECG) (a) and the histopathological injury (HI) (b) in isoproterenol- (ISO-) induced myocardial ischemia (MI) rat. The data are presented as means ± SD (*n* = 4).

The ECG result is shown in Figure 1(a). The elevated ST segment and abnormal *Q* wave in the ECG of MI group wereobserved and compared with those of the normal group. The ST-segment elevation (Figure 1(a) H) observed in the MI group, which represented the MI damage model, was established. However, after AAEs treatment, the ST segment in ECG was significantly decreased and the abnormal *Q* wave of ECG in MI rats did not appear, which suggests that the WTAAE had improved the ECG effect in MI rats induced by ISO.

### 3.2. Myocardium Histopathological Examination

To observe the damage degree of cardiac tissues, myocardial biopsies were macroscopically observed as shown in Figure 1(b). In the normal group, there was a thin and compact arrangement of striated muscle tissue in the heart tested by HE staining (Figure 1(b) A). However, the cardiac tissues in the MI group showed that most of the muscle fibers in the striated muscle tissue exhibited dissolution, degeneration, necrosis, and inflammatory cell infiltration and formed a large area of connective tissue overgrowth and bleeding (Figure 1(b) B). The above damage in the MI rats treated with AAEs obviously improved compared with those in the MI group (Figure 1(b) C–G). The effect of WTAAE was similar to WAAE and better than FBAAE under same dose.

### 3.3. Effect of AAEs on Apoptosis in Myocardial Tissue

**Figure 2:**
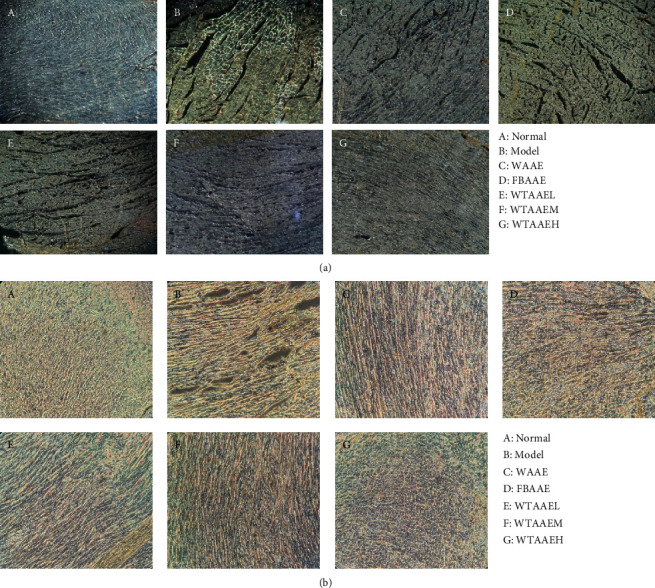
Effects of orally administered AAEs on the myocardial apoptosis under fluorescence observation (a) and myocardial apoptosis under optical observation (b) in isoproterenol- (ISO-) induced myocardial ischemia (MI) rat. The data are presented as means ± SD (*n* = 4).

The study in Section 3.2 showed that AAEs inhibited dissolution and necrosis in heart tissues. To assess whether AAEs affect myocardial apoptosis or not, we evaluated the myocardial apoptosis in MI induced by ISO (Figure 2). The TUNEL-positive expression significantly increased in the ischemic border of the myocardium in the MI group compared with that in the normal group (Figures 2(a) and 2(b) A-B), while AAEs treatment showed markable reduction of apoptosis compared with the MI group (Figures 2(a) and 2(b) C–G). TUNEL staining demonstrated that more apoptosis exhibited upon treatment with ISO, while AAEs inhibited apoptosis. Besides, the effect of WTAAE was similar to that of WAAE and better than that of FBAAE in 2.84 g/kg. These results suggest that AAEs exerts an antiapoptotic effect in MI mice.

### 3.4. Effect of AAEs on Myocardial Enzymes in Serum

**Figure 3:**
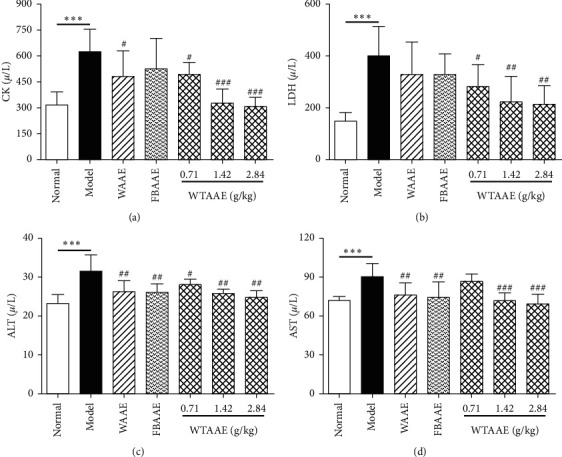
Effects of orally administered AAEs on the levels of CK (a), LDH (b), ALT (c), and AST (d) in the serum of isoproterenol- (ISO-) induced myocardial ischemia (MI) rat. The data are presented as mean ± SD (*n* = 6). ^*∗∗∗*^*P* < 0.001 vs. normal group; ^#^*P* < 0.05, ^##^*P* < 0.01, and ^###^*P* < 0.001 vs. model group indicate a significant difference.

As shown in Figures 3(a)–3(d), CK, LDH, ALT, and AST in the MI group rats were increased compared with those in the normal control rats (*P* < 0.001). After being treated with AAEs in MI rats, the serum concentration of CK, LDH, ALT, and AST was lower than that in MI rats (*P* < 0.05, *P* < 0.01, and *P* < 0.001). The effect of WTAAE was better than that of WAAE and FBAAE under the same dose; that is, WTAAE obviously improved MI induced by ISO.

### 3.5. Effect of AAEs on the Lipid Peroxidation in Plasma

**Figure 4:**
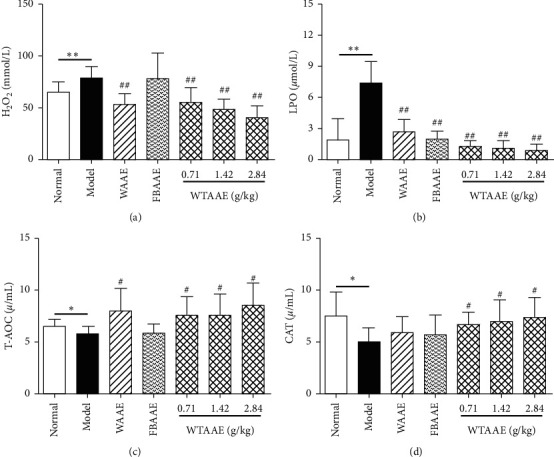
Effects of orally administered AAEs on the levels of H_2_O_2_ (a), LPO (b), T-AOC (c), and CAT (d) in heart tissues of the isoproterenol- (ISO-) induced myocardial ischemia (MI) rat. The data are presented as mean ± SD (*n* = 6). ^*∗*^*P* < 0.05 and ^*∗∗*^*P* < 0.01 vs. normal group; ^#^*P* < 0.05 and ^##^*P* < 0.01 vs. model group indicate a significant difference.

As shown in Figures 4(a)–4(d), the level of H_2_O_2_ and LPO were significantly increased (*P* < 0.01) in rats with MI compared to that of the normal rats, confirming that the oxidative damage occurred after ISO treatment. However, AAEs markedly decreased the level of H_2_O_2_ and LPO (*P* < 0.01) compared with that of the model group. In contrast, the level of T-AOC and CAT was significantly increased in WAAE (*P* < 0.05) and in the 0.71, 1.42, and 2.84 g/kg of WTAAE group (*P* < 0.05) as shown in Figures 4(c) and 4(d) compared to that in the model group, showing the potential antioxidative effect of AAEs.

### 3.6. Effect of AAEs on the Nrf2-ARE Signaling Pathway of Genes Expression in Heart Tissue

**Figure 5:**
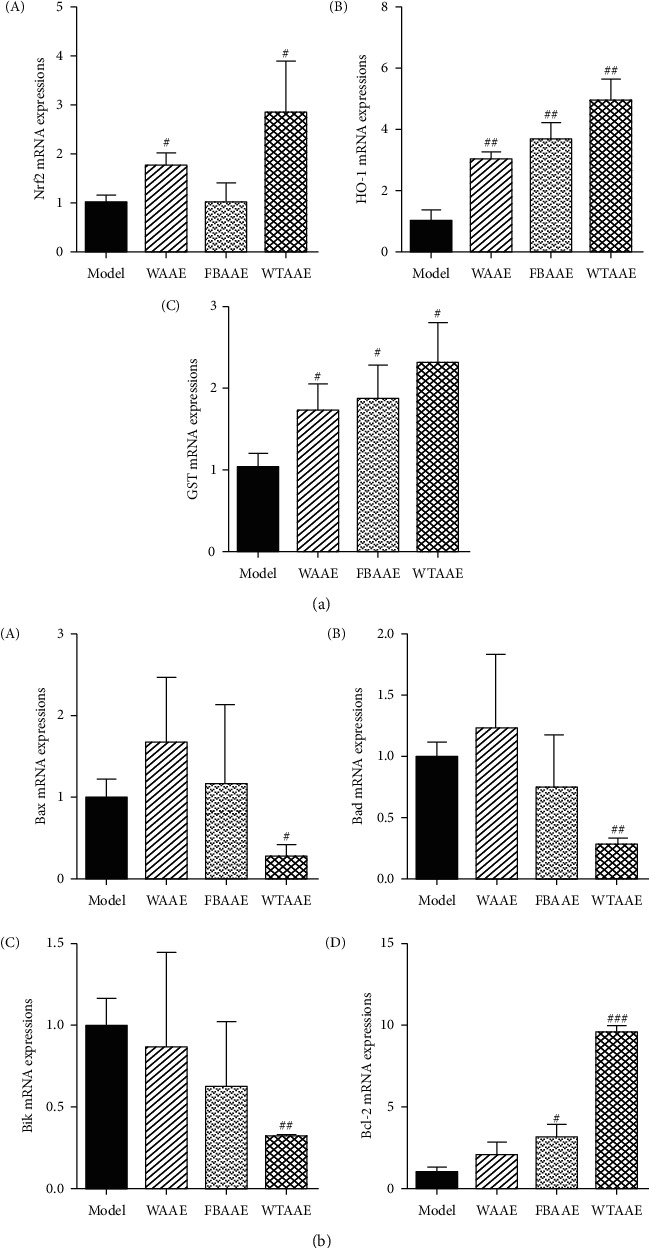
Effects of orally administered AAEs on the mRNA factors expression levels of Nrf2-ARE (a) and Bcl-2 family (b) signaling pathway in heart tissues of the isoproterenol- (ISO-) induced myocardial ischemia (MI) mice. The data are presented as mean ± SD (*n* = 3) of triplicate experiments. ^#^*P* < 0.05, ^##^*P* < 0.01, and ^###^*P* < 0.001 vs. model group indicate a significant difference.

In order to explore the protective mechanisms mediated by AAEs antioxidation in the MI induced by ISO, we further detected the mRNA expression of Nrf2-ARE pathway in heart tissue by using the RT-PCR. As shown in Figure 5(a), as expected, the treatment with WAAE and WTAAE significantly increased the mRNA expression levels of Nrf2 (*P* < 0.05) and AAEs significantly increased the levels of HO-1 and GST (*P* < 0.05 or *P* < 0.01) in heart tissue of ISO-induced MI rats, playing a significant antioxidant damage effect by upregulating and activating the Nrf2-ARE pathway.

### 3.7. Effect of AAEs on the Bax-Bcl-2 Signaling Pathway of Genes Expression in Heart Tissue

Next, in order to explore the protective mechanisms mediated by AAEs antiapoptosis in the MI induced by ISO, we also further detected the mRNA expression of Bax-Bcl-2 pathway by using the RT-PCR in heart tissue. As shown in Figure 5(b), it is particularly gratifying that WTAAE markedly decreased the mRNA expression levels of Bax, Bad, and Bik (*P* < 0.05 or *P* < 0.01) and increased the expression of Bcl-2 in the heart tissues, showing a significant antiapoptotic effect by regulating the Bax-Bcl-2 pathway.

## 4. Discussion and Conclusions

Agarwood, a precious traditional Chinese medicine, is used for centuries to treat several diseases, especially heart diseases [4, 24]. Isoproterenol (ISO), a synthetic catecholamine and *β*-adrenergic agonist, gives rise to acute irreversible myocardial ischemia injury in rats, once in an overdose situation [25, 26]. As it is widely accepted that ISO injection can readily induce acute MI in rats, and antioxidant activity is one of the key mechanisms of anti-MI efficacy [27]. Besides, we  previously  revealed  that the  components  of  WTAAE include sesquiterpenes  (10.615%), chromone  (31.678%), aromatics (31.831%), and  other  known  compounds  (25.760%) and chromone  had antioxidant and anti-inflammatory activities [28]. In the present study, we also verified MI induced by ISO in rat and first found that WTAAE could protect against MI as evidenced by biochemical parameters, ECG, and histological assessments. Furthermore, WTAAE also markedly inhibited the oxidative stress and played antiapoptotic effect, alleviating the ISO-induced MI injury, and the effect was better than that in WAAE and FBAAE. The mechanism may be correlated with the regulation of Nrf2-ARE and Bcl-2 family pathways.

MI injury results in serious acute or chronic myocardial damage, including myocardial ultrastructural alterations, remodeling, and systolic and diastolic dysfunctions, including ECG abnormality [5]. The relatively high content of CK, LDH, ALT, and AST of these enzymes in serum is used to diagnose and monitor MI accounts for the clinical usefulness. This is because leakage of these enzymes acted as a marker of myocardial cell membrane damage [29]. Our results showed that the abnormal ECG, ST-segment elevation, myocardial pathological injury, and myocardial enzyme elevation appeared in the model group. AAEs obviously reversed the abnormal ECG, relieved the pathological injury, and alleviated the degree of myocardia apoptosis. AAEs also decreased the levels of CK, LDH, ALT, and AST in serum of MI rats, which suggested that AAEs have obvious myocardial protective effect; especially the effect of 2.84 g/kg WTAAE was better than that of WAAE and FBAAE. Oxidative stress plays an essential role in the pathogenesis of MI injury. The major H_2_O_2_ and superoxide and hydroxyl radicals generated during ischemia injury [30, 31]. However, ROS are controlled by a system of enzymatic and nonenzymatic antioxidants such as SOD, CAT, GPx, and GSH, which scavenge free radicals [32]. T-AOC is used to characterize the total level of antioxidants [33]. However, a major mechanism of the defense against oxidative stress is the activation of NF-E2-related nuclear factor 2- (Nrf2-) antioxidant response element (ARE) signaling. Nrf2-ARE, a central controller, is responsible for diverse cytoprotective processes [34]. Under normal conditions, Nrf2 is localized in the cytoplasm by interaction with Kelch-like ECH associating protein 1 (Keap1), inhibiting Nrf2 ubiquitin-mediated degradation [35, 36]. Upon exposure to ROS or other stimuli, Keap1, degraded and activated, translocates into the nucleus and binds to the ARE, enhancing the production of many antioxidant enzyme genes [37, 38]. In the present study, our results showed that AAEs obviously enhanced the mRNA expressions of Nrf2, HO-1, and GST (Figure 5(a)); the content of T-AOC; and the activity of CAT (Figures 4(c) and 4(d)) and decreased the contents of H_2_O_2_ and LPO (Figures 4(a) and 4(b)) compared with those of the model group. AAEs had a significant effect on scavenging free radicals and antioxidative damage, in which the effect of WTAAE was greater than that of WAAE and FBAAE, indicating AAEs-played antioxidant effect and mediated myocardial protective effect by the upregulation of the Nrf2-ARE pathway.

A previous study showed that the mechanisms preconditioning myocardial apoptosis of MI may be connected with the increase of Bcl-2/Bax and the decrease of Bax [39]. Another study showed that Bcl-2 and Bax played an important role in MI and the repair process [40]. Therefore, the levels of Bcl-2 family were related to the decrease of heart tissue injury to some degree. The present study suggested that AAEs significantly decreased the mRNA expressions of Bax, Bad, and Bik, increased the level of Bcl-2 (Figure 5(b)), particularly the WTAAE group, contributing to the antiapoptotic effect of MI by controlling the occurrence of apoptosis in heart tissues.

In this study, our results firstly confirmed that the myocardial protection of WTAAE in MI, with the effect, was better than that of WAAE and FBAAE. From these results, we conclude that the potential mechanisms are related to antioxidative and antiapoptotic effects. However, the specific mechanism of this action needs to be further confirmed. Generally, our study suggested that agarwood could be as a potential protection drug in the prevention and therapy of cardiac disease.

## Figures and Tables

**Table 1:** Primer sequences used in RT-PCR.

Genes	Primer sequences
Nrf2	Forward 5′-TCTTGGAGTAAGTCGAGAAGTGT-3′
	Reverse 5′-GTTGAAACTGAGCGAAAAAGGC-3′
HO-1	Forward 5′-AAGCCGAGAATGCTGAGTTCA-3′
	Reverse 5′-GCCGTGTAGATATGGTACAAGGA-3′
	Forward 5′-TGCCCAAGTCCACGAATACC-3′
	Reverse 5′-CCATTCTATCTCTGTTCCGTTCC-3′
GST	Forward 5′-CCGGCGAATTGGAGATGAACT-3′
	Reverse 5′-CCAGCCCATGATGGTTCTGAT-3′
Bad	Forward 5′-CTCCGAAGGATGAGCGATGAG-3′
	Reverse 5′-TTGTCGCATCTGTGTTGCAGT-3′
Bik	Forward 5′-ACGTGGACCTCATGGAGTG-3′
	Reverse 5′-TGTGTATAGCAATCCCAGGCA-3′
Bcl-2	Forward 5′-GTCGCTACCGTCGTGACTTC-3′
	Reverse 5′-CAGACATGCACCTACCCAGC-3′
*β*-actin	Forward 5′-GGCTGTATTCCCCTCCATCG-3′
	Reverse 5′-CCAGTTGGTAACAATGCCATGT-3′

## Data Availability

The data used to support the findings of this study are available from the corresponding author upon request.
